# DUF581 Is Plant Specific FCS-Like Zinc Finger Involved in Protein-Protein Interaction

**DOI:** 10.1371/journal.pone.0099074

**Published:** 2014-06-05

**Authors:** Muhammed Jamsheer K, Ashverya Laxmi

**Affiliations:** National Institute of Plant Genome Research, New Delhi, India; Leibniz-Institute for Vegetable and Ornamental Plants, Germany

## Abstract

Zinc fingers are a ubiquitous class of protein domain with considerable variation in structure and function. Zf-FCS is a highly diverged group of C_2_-C_2_ zinc finger which is present in animals, prokaryotes and viruses, but not in plants. In this study we identified that a plant specific domain of unknown function, DUF581 is a zf-FCS type zinc finger. Based on HMM-HMM comparison and signature motif similarity we named this domain as FCS-Like Zinc finger (FLZ) domain. A genome wide survey identified that FLZ domain containing genes are bryophytic in origin and this gene family is expanded in spermatophytes. Expression analysis of selected *FLZ* gene family members of *A. thaliana* identified an overlapping expression pattern suggesting a possible redundancy in their function. Unlike the zf-FCS domain, the FLZ domain found to be highly conserved in sequence and structure. Using a combination of bioinformatic and protein-protein interaction tools, we identified that FLZ domain is involved in protein-protein interaction.

## Introduction

Identifying gene function and their interaction with other genes with respect to the regulation of growth and development is major task post genome sequencing. Although *Arabidopsis thaliana* genome sequencing was completed in late 2000, the functions of a large number of genes are still unknown [1], [2]. According to TAIR10, out of 27,416 protein coding genes in *A. thaliana*, functions of about 37% genes are unidentified [2]. To further complicate this issue, many uncharacterized and even some functionally characterized proteins contain domains whose function is unknown. These uncharacterized domains are known as Domains of Unknown Functions (DUFs). DUF nomenclature was introduced to record and classify the conserved domains which are present in proteins while no information about its function was available at that time. The number of DUFs is so huge; PFAM release 23.0 include over 2200 protein families of DUFs which cover almost 22% of the total PFAM protein families [3]. It is presumed that majority of DUFs are divergent members of the already existing domains and the rest can be novel folds. Although the numbers of DUF families are increasing in PFAM, the identification of functions of DUF domains is slowly gaining momentum. The DUF3233 of gram negative gamma proteobacteria found to be trans-membrane β-barrel domain of auto-transporter proteins [4]. The DUF283 of Dicer endonuclease is predicted to form a double-stranded RNA-binding fold [5]. Later, structural analysis proved that DUF283 form a noncanonical double-stranded RNA-binding fold and functional studies confirmed that it has a weak double strand RNA binding activity and a specific protein binding activity [6]. The co-ordinated effort of NIH Protein Structure Initiative identified the structures of about 250 DUFs and found that majority of them are divergent members of the well characterized domains [7].

DUF581 is a plant specific domain found in all taxa except algae. They are highly conserved across plant kingdom and least explored. An *A. thaliana* DUF581 containing protein, MEDIATOR OF ABA-REGULATED DORMANCY 1 (MARD1) was identified from senescence related enhancer-trapping and found to be involved in ABA-mediated seed dormancy and induced during senescence [8], [9]. They also identified that MARD1 possess a novel zinc finger domain suggesting the relation of DUF581 with zinc fingers of bacteria, archaea and metazoans [9]. A large scale protein-protein interaction study in *A. thaliana* identified many interacting proteins of DUF581 family proteins; however, the biological significance of these interactions remains to be explored [10].

DUF581 show high signature motif similarity with MYM-type Zinc finger with FCS sequence motif (zf-FCS). Zf-FCS is first identified in MYM family proteins which are related to myeloproliferative syndrome and mental retardation [11]. They are present in viruses, eubacteria, archaea, metazoa but not in plants. One FCS type zinc finger protein is present in brown algae *Ectocarpus siliculosus*. Zf-FCS is named after the conserved phenyl alanine and serine residues associated with the third cysteine. In metazoans, zf-FCS is largely present in Polycomb-group (PcG) of proteins. PcG proteins are developmental-regulator proteins which silence the expression of downstream proteins through chromatin-remodeling and epigenetic silencing. They form a multi-protein Polycomb Repressive Complex (PRC) which bind to the target gene and alter the epigenetic status of the gene [12]. PcG proteins are first identified in *Drosophila melanogaster* for silencing the expression of HOX genes which is important in proper embryonic-development [13]. They are highly conserved regulatory proteins which play an important role in regulating developmental events in plants and animals [14]. Zf-FCS is found as single domain or in tandem cluster of up to 10 repeats. Only few studies are done related to this domain which proved that it is a diverse class of zinc finger with variable functions. The single zf-FCS in Rae28, mouse homologue of *D. melanogaster* Polyhomeotic protein, interacts with RNA and DNA in non-sequence-specific manner [15]. Since Rae28 is involved in chromatin-remodeling, it is hypothesized that this zinc-finger may be involved in the binding of PRC complex to the target sequence. Later, it is found that the direct interaction of zf-FCS domain of Human Polyhomeotic Homologue 1 (HPH1/PHC1) with RNA is required for PHC-mediated repression of target genes [16]. Zf-FCS domain of human dSfmbt homologue L (3) MBT-like 2 (L3MBTL2) is a treble clef zinc finger similar to zinc fingers involved in protein-nucleic acid interaction [17]. These results suggest that zf-FCS is involved in protein-nucleic acid interaction. However, it is also reported that zf-FCS is involved in protein-protein interaction. It is found that the direct interaction among *D. melanogaster* PcG proteins, Scm-related protein containing four mbt domains (dSfmbt) and Sex comb on midleg (Scm) is mediated by the zf-FCS domains present in both proteins. Both these proteins interact and cooperate synergistically for mediating target gene repression [18]. All these reports shows that zf-FCS is a structurally diverse family which accommodate both nucleic-protein and protein-protein interaction zinc fingers.

This study aims to characterize the function of DUF581 protein domain which is exclusive to plants. Using sensitive bioinformatic approaches, we confirmed that DUF581 is a zf-FCS like zinc finger domain. We named this plant specific domain as FCS-Like Zinc finger (FLZ). A genome wide survey identified that FLZ domain has a bryophytic origin and this gene family is expanded in higher plants. Phylogenetic analysis of *A. thaliana* FLZ domain proteins and expression analysis of selected *FLZ* genes are done. Sequence and structure conservation studies identified that unlike the zf-FCS domain, FLZ domain is highly conserved. FLZ domain predicted to form a novel alpha-beta-alpha secondary structure pattern. A combination of bioinformatics and protein-protein interaction tools identified that FLZ acts as a protein-protein interaction module.

## Results

### DUF581 Domain Containing Proteins are Plant Specific FCS-Like Zinc Finger Proteins

A genome wide survey was conducted in different databases to identify the members of DUF581 domain containing proteins from sequenced plant genomes. 331 members were identified from PFAM and 474 members were identified from InterPro [3], [19]. Genes were also identified from Phytozome, Plaza, NCBI, Solanaceae Genomic Resource at Michigan state university, Tomato Genome Database at MIPS and ConGenIE [20]–[24]. Sequences were manualy curated to remove repeats and outliers. The conservation at signature motif and structural conservation were verified. PFAM identified a DUF581 domain containing protein from a parasitic heterokont, *Blastocystis hominis*; however, in our analysis we found that this domain lackedthe conserved alpha-beta-alpha structural pattern specific to the plant DUF581 domain. A total of 757 non-redundant DUF581 genes were identified from 41 plant genomes (Table 1). DUF581 gene family is plant specific excluding algae. Search in *Ostreococcus tauri, O. lucimarinus, Micromonas sp. RCC299, Volvox carteri, Chlamydomonas reinhardtii* genomes found no hits suggesting that DUF581 genes were absent in algae. All members of viridiplantae contains DUF581 domain containing genes. *Physcomitrella patens* genome contains 2 DUF581 genes suggesting a bryophytic origin of this gene family. Pteridophyte, *Selaginella moellendorffii* also possess 2 DUF581 genes. Spermatophytes show an increased content of DUF581 genes ranging from 9 members in *Capsicum annum, Carica papaya, Aquilegia caerulea* and *Lotus japonicus* to *48* in *Panicum virgatum*. A detailed list of all DUF581 proteins identified in this study is given in Table S1.

**Table 1:** Distribution of *FLZ* gene family in sequenced genomes.

Taxonomic position	Species	Number of *FLZ* genes
Bryophyta	*Physcomitrella patens*	2
Pteridophyta	*Selaginella moellendorffii*	2
Gymnosperms	*Picea abies*	23
Dicots	*Arabidopsis thaliana*	18
	*Arabidopsis lyrata*	18
	*Aquilegia caerulea*	9
	*Brassica rapa*	34
	*Capsella rubella*	16
	*Capsicum annum*	9
	*Carica papaya*	9
	*Cicer arietinum*	15
	*Citrus clementina*	13
	*Citrus cinensis*	13
	*Cucumus sativus*	16
	*Eucalyptus grandis*	19
	*Fragaria vesca*	14
	*Glycine max*	37
	*Gossypium raimondii*	28
	*Linum usitatissimum*	16
	*Lotus japonicus*	9
	*Malus domestica*	22
	*Manihot esculenta*	18
	*Medicago truncatula*	12
	*Mimulus gluttatus*	14
	*Nicotiana tabacum*	25
	*Phaseolus vulgaris*	19
	*Populus trichocarpa*	21
	*Prunus persica*	12
	*Ricinus communis*	11
	*Solanum lycopersicum*	15
	*Solanum phujera*	15
	*Thellungiella halophila*	16
	*Theobroma cacao*	12
	*Vitis vinifera*	10
Monocots	*Brachypodium distachyon*	26
	*Hordeum vulgare*	16
	*Oryza sativa*	29
	*Panicum virgatum*	48
	*Setaria italica*	28
	*Sorghum bicolor*	29
	*Zea mays*	29

**Figure 1:**
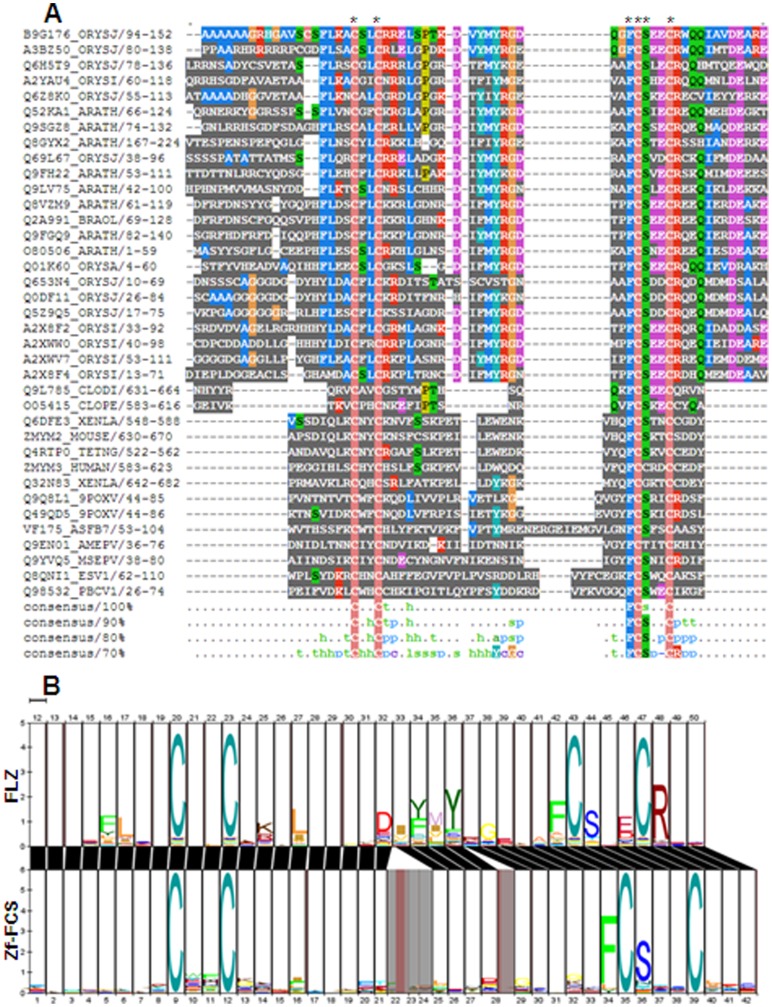
Alignment between FLZ and zf-FCS domain. (A) Multiple sequence alignment between FLZ and zf-FCS domains. Conserved cysteine, phenyl alanine and serine residues are marked by asterisks. (B) HMM-HMM alignment between FLZ domain and zf-FCS showing similarity in sequence conservation.

DUF581 and zf-FCS domain are members of TRASH clan of PFAM database and show very high similarity in sequence conservation (Figure S1). TRASH super family includes cysteine co-ordinated metal binding group of domains conserved both in prokaryotes and eukaryotes [25]. The other members of this super family include MYND, mitochondrial splicing suppressor 51, HIT zinc fingers, two DUF domains DUF2256 and DUF329, metal-binding domains archaeal TRASH domain, putative metal-binding domain of cation transport ATPase, YHS domain, and ribosomal protein L24e. All the members of TRASH clan shows varying degree of similarity in signature sequence motif (Figure S1). Sequence alignment between metazoan zf-FCS domains and DUF581 domains from plants shows that they possess very similar consensus cysteine-signature sequence with conserved phenyl alanine and serine residue associated with third cysteine (Figure 1A). Zf-FCS possess consensus CX_2_CX_14–30_FCSX_2_C zinc finger motif while DUF581 shows identical CX_2_CX_17–19_FCSX_2_C motif. In HMM-HMM comparison, both domains show a very similar alignment suggesting that both domains are nearly identical in signature sequence motif (Figure 1B). The above results suggest that DUF581 is a zf-FCS like C2-C2 zinc finger. Based on these observations, we named DUF581 as FCS-Like Zinc finger (FLZ) domain. The proteins which possess this domain are named as FCS-like zinc finger (FLZ) proteins.

### The *Arabidopsis FLZ* Gene Family

*A. thaliana* genome possesses 18 FLZ domain genes (Table 1). Except *AT3G63230*, all other genes have only single splice form while *At3g63230* forms two splice variants. *AT1G53885* and *AT1G53903* were found to be tandem duplicates and possess exactly same gene sequence. To understand the evolutionary relationship between individual members, a phylogram was constructed using the full length protein sequence of all FLZ proteins (Figure S2). The phylogram distinguished different clades of FLZ proteins. On the basis of their relation with FLZ1 observed in phylogram, all the other members were named. Among all the proteins, FLZ16 and FLZ17/18 showed most divergence from other members and formed individual distinct clades. Similarly, FLZ15 also formed a distinct clade from other proteins. All other members were grouped in two big clades representing 7 members each in clade I and II. Few members in each clade were very closely positioned hinting the possible redundancy in their function. Redundancy in expression pattern and function is a common feature observed in many multigene families of *A. thaliana* [26], [27]. Analysis of expression profile of three closely related members of *FLZ* gene family from clade I from publically available microarray data revealed that they show both distinct and overlapping expression pattern (Figure S3). The maximum expression of *FLZ1* was observed in the developing seeds. *FLZ2* and *FLZ3* were also fairly expressed in different seed stages. Apart from seed stages, *FLZ1* showed higher expression in imbibed seeds, stamens, carpels, and transition shoot apex while *FLZ2* is profusely expressed in cauline leaf, first node, and second internode and in different floral stages and organs. *FLZ3* had almost uniform expression pattern which profuse up regulation in 1^st^ node, 2^nd^ internode, cotyledon, and in different floral organs. *FLZ1*, *FLZ2* and *FLZ3* were also showed higher expression in senescing leaves compared to rosette leaves.

### FLZ Domain is a Novel Zinc-finger Domain with a Highly Conserved Alpha-beta-alpha Secondary Structure Pattern

**Figure 2:**
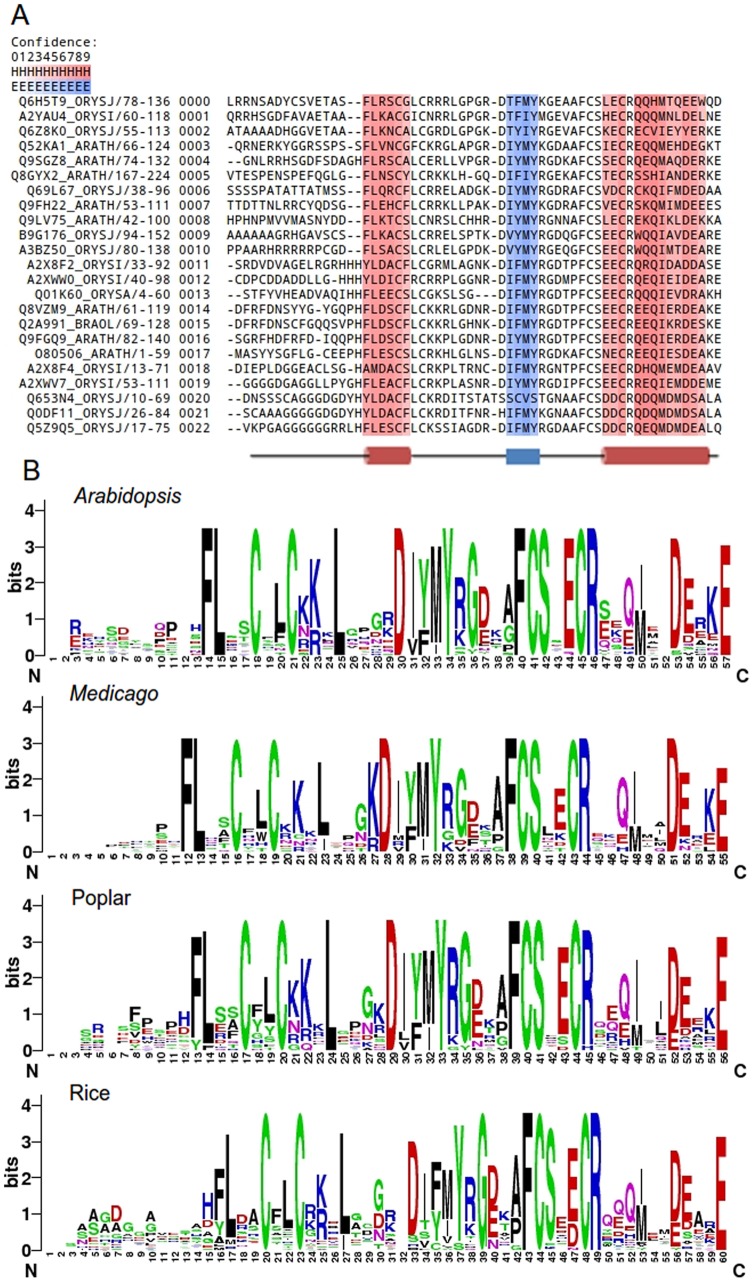
Secondary structure pattern and sequence conservation of FLZ domain. (A) Secondary structure conservation of FLZ domain. Red color indicates alpha helix and blue color indicates beta-sheet. Confidence gradient of secondary structure formation is given on the top. (B) Sequence logo of *Arabidopsis*, *Medicago,* poplar and rice FLZ domains showing amino acid conservation.

FLZ domain predicted to have a highly conserved secondary structure pattern. It composed of an N-terminal short α-helix, a beta hairpin followed by a longer C-terminal α-helix (Figure 2A). Interestingly, this kind of secondary structure pattern is not found in any of the classified structural classes of zinc fingers [28]. Residue conservation analysis in the FLZ domain across plant kingdom showed that the four cystein residues are highly conserved along with signature phenyl alanine and serine residues associated with third cysteine (Figure 2B). It has a highly conserved α helix- β hairpin- α-helix secondary structure pattern as a result of conserved amino acids which favors the formation of α-helix and β-sheet at the specific regions. Alanine, cysteine, leucine, methionine, lysine, glutamine and histidine show high helix forming propensity while tyrosine, valine, phenyl alanine, isoleucine, tryptophan, and threonine favor beta sheet [29], [30]. The highly conserved phenyl alanine and lysine residues followed by fairly conserved aspartic acid and alanine along with the first cysteine and the following phenyl alanine contribute to the formation of the N-terminal short helix. In helices, glutamic acid, phenyl alanine and aspartic acid are found in larger frequencies than expected according to their helix-propensity [29]. The middle beta-sheet is formed by the conserved isoleucine, phenyl alanine, methionine, and tyrosine residues. The larger C-terminal helix is in the position of fourth cysteine associated with conserved glutamic acid and fairly conserved arginine, aspartic acid, and glutamine residues which generally favors helix formation. Along with the highly conserved cysteine residues, the fair conservation of the other residues resulted in a highly conserved topology of FLZ domain across the plant kingdom.

### Domain Organization and Distribution in FLZ Protein Family

**Figure 3:**
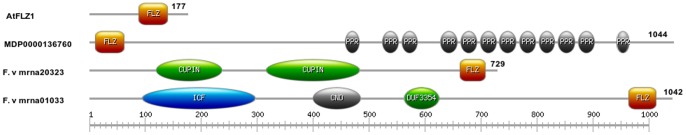
Schematic representation of domain organization in FLZ protein family. The FLZ proteins are scanned by InterProScan to identify conserved domains. *A. thaliana* FLZ1 is shown as a representative model for proteins which contain FLZ domain only. Proteins which possess other domains along with FLZ are also shown. The domains are abbreviated as follows, FLZ (FCS like zinc finger, PF04570), PPR (Pentatricopeptide Repeat, PF01535), Cupin (Cupin 1, PF00190), ICF (Ion channel family, PF00520), CND (Cyclic nucleotide-binding domain, PF00027) and DUF3354 (Domain of unknown function 3354, PF11834).

Domain distribution and organization of FLZ family proteins were analyzed by InterProScan [31]. Except three members, all other members contain no other functional domain other than FLZ, suggesting the pivotal role of FLZ domain in their function (Figure 3). In most cases, the single FLZ domain is situated near the C-terminal end of the protein. Two *Fragaria* proteins contain other domains along with FLZ domain. F.ve mrna20323.1 contains two Cupin (PF00190) domains while F.ve mrna01033.1 contains an ion-transport protein domain (PF00520), a cyclic nucleotide-binding domain and DUF3354 (PF11834) along with a C-terminal FLZ domain. A FLZ protein in apple, MDP0000136760, shows tandem pentatricopeptide repeats along with an N-terminal FLZ domain.

### FLZ Domain is Involved in Protein-protein Interaction

Threading/fold recognition is helpful in identifying structural and functional aspects of novel folds even if they possess remote homology with characterized domains [32], [33]. Threading of FLZ with Phyre revealed that it shows high fold similarity with LIM domains (Figure S4). LIM domains are zinc finger domains with two tandem zinc fingers. Each of these zinc fingers forms a treble-clef fold and participates in protein-protein interaction [34]. Threading of FLZ gave reliable predictions with a precision up to 90% for LIM domains. This prompted us to speculate that FLZ might also be a protein-protein interaction zinc finger.

**Figure 4:**
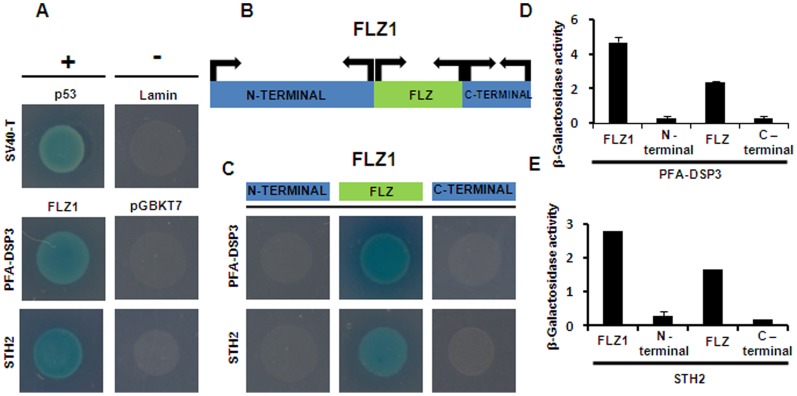
FLZ acts as the module for protein-protein interaction. (A) FLZ1 interacts with PFA-DSP3 and STH2 in Y2H. Murine p53 and SV40 large T-antigen interaction taken as positive control and p53 and lamin interaction is taken as negative control. (B) The deletion constructs of FLZ1, N-terminal (1–88 amino acids), FLZ (89–140 amino acids) and C-terminal (141–177 amino acids). (C) Y2H with deletion constructs of FLZ1 with PFA-DSP3 and STH2 showing FLZ is essential for their interaction. (D) And (E) beta-galactosidase activity of full length FLZ1 and deletion constructs interaction with PFA-DSP3 and STH2.

To find out whether FLZ protein involved in protein-protein interaction, yeast-two-hybrid assay (Y2H) was conducted with an *A. thaliana* FLZ domain containing protein, AT5G47060. We named this protein as FCS-like Zinc Finger 1 (FLZ1). 50 colonies screened to identify the interacting proteins and 4 genuine interacting proteins are identified. A list of all interacting proteins identified in this study is given in Table S2. To find out whether the FLZ domain of FLZ1 is involved in protein-protein interaction, deletion constructs of *FLZ1* gene were generated (Figure 4B). The N terminal fragment corresponds to 1 to 88 amino acids of the full length FLZ1 protein while the FLZ domain corresponds to amino acids from 89 to 140. The C-terminal fragment comprised of amino acids from 141 to 177 of whole protein. We repeated the Y2H with deletion fragments of FLZ1 with PLANT AND FUNGI ATYPICAL DUAL-SPECIFICITY PHOSPHATASE 3 (PFA-DSP3) and SALT TOLERANCE HOMOLOG2 (STH2) which are earlier found to be interacting with full-length FLZ1 (Figure 4A). In Y2H with deletion constructs, we found that only FLZ domain can mediate the protein-protein interaction with the prey proteins suggesting their role in protein-protein interaction (Figure 4C). In beta-galactosidase assay, FLZ domain showed nearly half strength of interaction compared to full length bait while N-terminal and C-terminal fragments showed very minimal enzyme activity proving that FLZ domain alone is responsible for interaction of FLZ1 with other proteins (Figure 4D, E). However, the strength of the interaction is reduced to almost half when FLZ domain alone interacted with prey proteins suggesting that the other parts of the protein may be helping in providing a strong interaction between both proteins.

**Figure 5:**
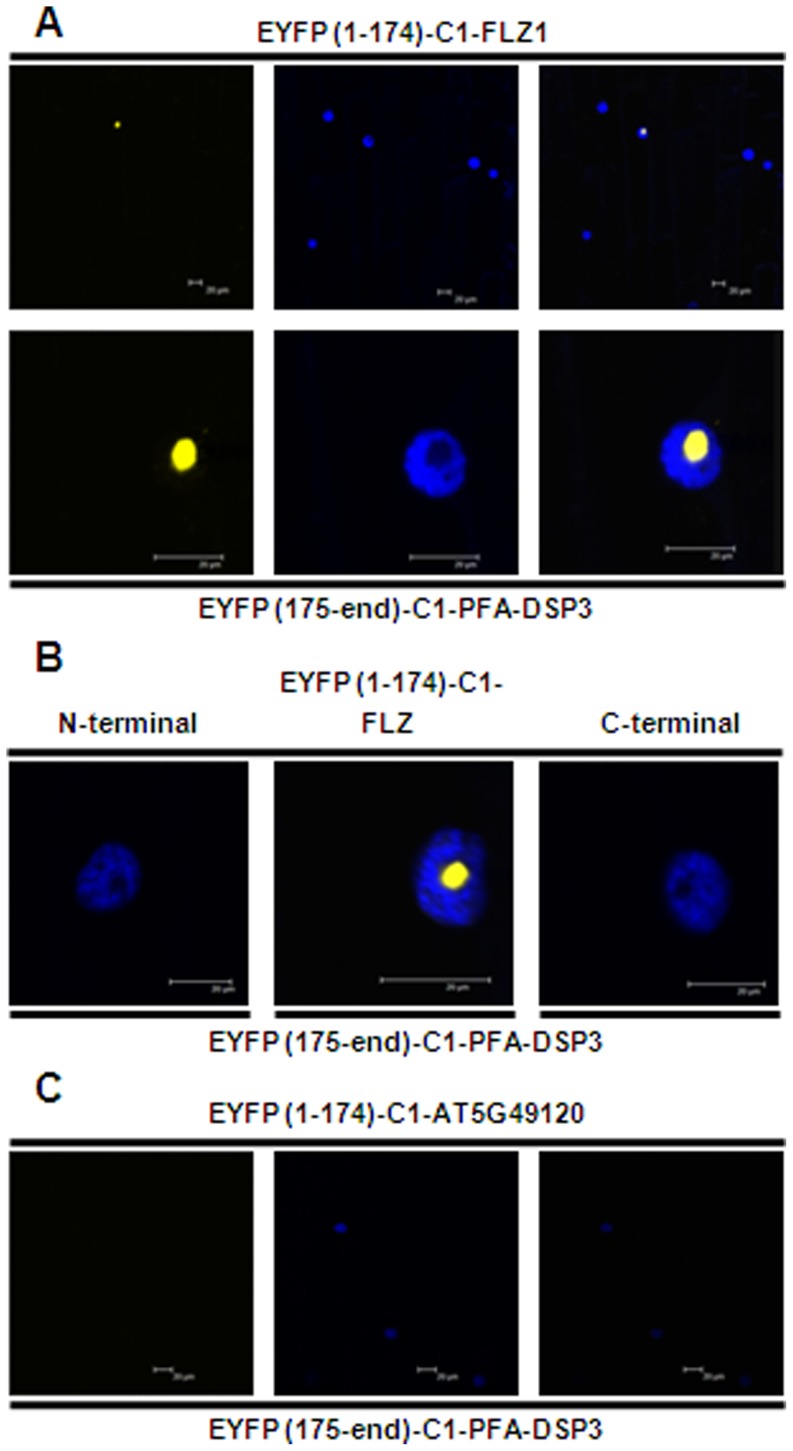
FLZ domain mediates the interaction of FLZ1 and PFA-DSP3. (A) BiFC, FLZ1 and PFA-DSP3 interact exclusively in nucleolus. Upper panel: whole cell, YFP alone, DAPI alone and merged. Lower panel: nucleus zoomed, YFP alone, DAPI alone and merged. (B) BiFC of PFA-DSP3 with deletion constructs of FLZ1: N-terminal (1–88 amino acids), FLZ (89–140 amino acids) and C-terminal (141–177 amino acids). (C) BiFC of PFA-DSP3 with AT5G49120: YFP alone, DAPI alone and merged. YFP were excited at 514 nm and emission was recorded at 530 nm. DAPI were excited at 351 nm and emission was recorded at 450 nm.

**Figure 6:**
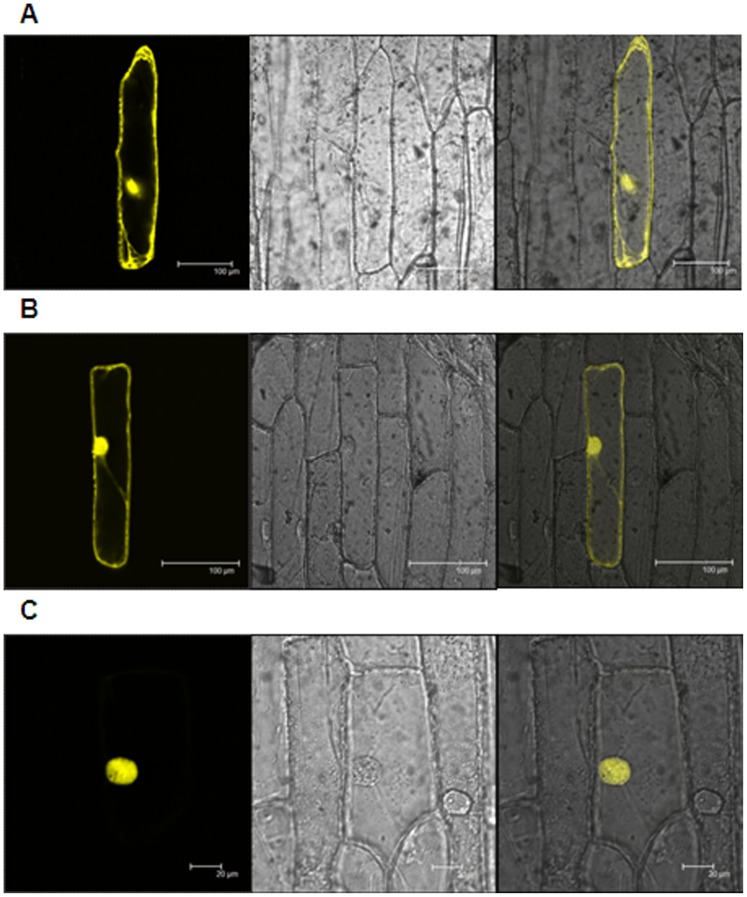
Sub-cellular localization of FLZ1 and PFA-DSP3 in onion epidermal cells. (A) Vector alone. (B) FLZ1. (C) PFA-DSP3. Left to right, YFP only, bright field, merged. FLZ1 is localized in both nucleus and cytoplasm while PFA-DSP3 is exclusively localized in nucleus. YFP were excited at 514 nm and emission was recorded at 530 nm.

To confirm the results obtained from Y2H assay, we did BiFC assay of FLZ1 and PFA-DSP3 interaction. In BiFC assay using onion epidermis system, it was found that both these proteins interact in the nucleolus (Figure 5A). Apart from its wide use as a DNA stain, DAPI is also used as a negative stain for nucleolus [35]–[37]. Negative staining of nucleolus with DAPI confirmed that both proteins interact exclusively in the nucleolus (Figure 5A). Further, we checked whether FLZ domain alone can mediate the interaction between FLZ1 and PFA-DSP3. As observed in the Y2H experiment, we found that FLZ domain is alone sufficient for the interaction of both these protein confirming the role of FLZ domain in protein-protein interaction (Figure 5B). To confirm the specificity of this interaction, we used another *A. thaliana* FLZ domain containing protein, AT5G49120 and checked whether it can interact with PFA-DSP3. It was found that AT5G49120 cannot interact with PFA-DSP3 suggesting that the interaction is very specific to FLZ1 (Figure 5C). Normally, FLZ1 localizes in nucleus and cytoplasm while PFA-DSP3 localizes exclusively in nucleus (Figure 6). However, their interaction found to be exclusive to nucleolus suggesting a possible role in nucleolar function.

## Discussion

In this study we identified FLZ domain containing proteins are identified from 41 plant species. They are completely absent in algae. The first report of FLZ domain proteins came from bryophyte, *P. patens* suggesting a bryophytic origin. In higher plants, the *FLZ* gene family is highly expanded. Most of the plants are paleopolyploids. Two whole genome duplication events happened before the diversification of seed plants expanded and diversified many of the regulatory gene families, especially genes which are related to flowering and seed development [38]. Gene families are evolved from segmental and tandem gene duplication of parent genes [39]. Most number of *FLZ* genes are found in the tetraploid genome of *P. virgatum* AP13, implying the role of genome duplication in expansion of *FLZ* gene family.

Analysis of evolutionary relationship between *Arabidopsis FLZ* proteins revealed the position of individual members inside the family. Expression profiling of three closely related members revealed an overlap in their expression domain suggesting the possible redundancy in function. In general, all three proteins were expressed in different floral organs, flower and seed developmental stages. *FLZ1* was also expressed in transition shoot apex suggesting a role in regulating phase transition. In Y2H, we identified that FLZ1 interact with CONSTANS-LIKE 1 (COL1), which is a homologue of flowering time gene CONSTANS (CO). FLZ1 also interacts with STH2 which is mainly involved in light regulated development and shade avoidance [40], [41]. We identified that FLZ1 interact with a dual specificity phosphatase, PFA-DSP3 in nucleolus. Identification of biological significance of these interactions can shed light to the possible role of FLZ1 in different developmental stages. As like *MARD1*, all three genes analyzed in this study showed transcript accumulation in senescing leaves compared to rosette leaves suggesting the function of *FLZ* gene family in senescence.

*FLZ* genes are a poorly studied class of gene family which is specific to plants. Early efforts in understanding the role of these genes identified that they are related to senescence and ABA mediated seed dormancy [8], [9]. They are small proteins and almost all of them contain only a single FLZ functional domain. Decoding the function of FLZ is a key for the functional characterization of this family. From the individual functional characterization of DUF families and the co-ordinated work of NIH Protein Structure Initiative, it is found that most of the DUFs are the diverged members of the already characterized domains [4], [7], [42]. Taking this notion in account, the analysis of sequence conservation of FLZ domain clearly identified that they are highly related to zf-FCS. As in the case of zf-FCS, the phenyl alanine and serine residue associated with third cysteine is also fairly conserved in FLZ domain. The major difference between both these domains is in the length of the spacer region which connects the zinc repeats. The spacer region of zf-FCS is highly variable with residues from 14 to 30. However, the spacer region of FLZ is much conserved with residue variation from 17 to 19 only. It is already found that the spacer region of zinc fingers varies even among the members of the same class and the variation in the spacer region influences the function of the zinc finger [43], [44]. It is evident that the divergent functions played by zf-FCS are because of the variation in the length of spacer region. This variation resulted in different secondary structure pattern which makes zf-FCS as a multifunctional zinc finger class (Data not shown). However, in the case of FLZ domain, the variation in the spacer length is only two residues suggesting a highly conserved function across the species.

In case of identifying the function of DUF, structure based approach is found to be more effective than sequence based search. The function of a protein domain is defined by the fold it forms, so during the course of evolution the structure is likely to be more conserved than the sequence [45]. Identification of the structure of the DUF and searching the close fold from already solved structures helped in identifying the function of many DUF domains [6], [7], [42]. Fold recognition can also be employed for identifying the homology of DUF with already solved structures. The fold recognition of FLZ domain identified that they are structurally very similar to LIM domain protein which is a protein-protein interaction zinc finger. Subsequently, we found that the FLZ domain of *A. thaliana* FLZ1 protein is indispensable for its interaction with PFA-DSP3 and STH2. However, the strength of the interaction is reduced to half when FLZ domain alone interacted with PFA-DSP3 and STH2 which suggests that the other portions of the protein might be having a helping role in ensuring a tight interaction. Notably, the FLZ is not structurally similar to the protein-protein interaction zf-FCS domains of dSfmbt and Scm (Data not shown). All these results suggest that FLZ domain is a highly diverged group of plant specific zf-FCS which functions as a protein-protein interaction module.

The analysis of secondary structure pattern identified that FLZ form an alpha-beta-alpha secondary structure pattern. Interestingly, this kind of secondary structure pattern is not reported in any classified zinc finger groups so far [28]. It is also observed that unlike zf-FCS domain, the FLZ domain is highly conserved in sequence and structure. Considering the conservation in structure and its relation with LIM domain, it is unlikely that FLZ domain also interact with nucleic acids as like some members of zf-FCS. The variation in the sequence and structure in the zf-FCS group must be the reason for their diverse functions such as nucleic acid binding and protein binding. A structure based classification of zf-FCS will be helpful to differentiate the functional subclasses and to understand the evolution of this divergence.

In short, using a combination of bioinformatics and protein-protein interaction studies, we found that DUF581 is FCS-like zinc-finger which acts as module for protein-protein interaction. They possess a highly conserved and novel secondary structure pattern. FLZ domain containing proteins are plant specific and bryophytic in origin. Local and whole genome duplication resulted in the expansion of this gene family in higher plants. Expression analysis of selected *A. thaliana FLZ* gene family members showed an overlap in the expression domain.

## Materials and Methods

### Identification of FLZ Gene Family Members from Public Data Bases

In this study, we identified *FLZ* family genes from 41 species of viridiplantae. Using the key word ‘DUF581’, a search was performed in PFAM, PLAZA v 2.5 and Interpro [3], [21], [19]. Genes were also identified from Phytozome using PFAM identifier, PF04570 [20]. *FLZ* genes from Solanaceae were identified from Solanaceae Genomic Resource using InterPro id IPR007650. Members from barley and *Cicer arietinum* were identified from NCBI BLASTp [22]. The *Picea abies FLZ* genes were identified from ConGenIE using BLASTp [24]. Protein sequence were downloaded and manually curated for repeats. Outliers were removed using InterProScan and multiple sequence alignment using Clustal X 2.0 [31], [46]. The structural conservation was analyzed using Ali2D [47].

### Bioinformatics Tools Used

For multiple sequence alignment, FLZ and zf-FCS domain sequences were retrieved from PFAM. They were aligned with Clustal X 2.0 and visualized using Mview [46], [48]. Pair wise HMM logo comparison was done using LogoMat-P [49]. Fold recognition of FLZ domain was done using Phyre v 0.2 [50]. Sequence logo was generated using WebLogo [51]. The domain organization was drawn by PROSITE My Domains [52]. The phylogenetic tree of *Arabidopsis FLZ* gene family was generated using MEGA 5 [53]. The expression graphs of *FLZ* genes were obtained from *Arabidopsis* eFP browser [54].

### Yeast Two-hybrid Assay

Yeast two-hybrid assay was conducted using Matchmaker Gold Yeast two-hybrid System (Clontech, Mountain View, CA) according to manufacturer’s protocol. *FLZ1* was cloned in pGBKT7 and used as a bait to screen normalized Mate & Plate Universal *Arabidopsis* Yeast two-hybrid cDNA library (Clontech, Mountain View, CA). The interaction of PFA-DSP3 and STH2 was confirmed by cloning them in pGDAT7 and one-to-one interaction check with FLZ1. pGBKT7-53 and pGADT7-T were used as positive control and pGBKT7-Lam and pGADT7-T were used as negative control for the experiments. Deletion constructs of FLZ1 was made in pGBKT7 and interaction was checked with pGDAT7-PFA-DSP3 and pGDAT7-STH2. The primers used for cloning are shown in Table S3.

### β-Galactosidase Assay

Bait and prey proteins were co transformed in Y187 yeast strain and β-Galactosidase assay was conducted according to the protocol of Yeast Protocols Handbook (Clontech, Mountain View, CA). The result was the average of three independent experiments.

### Bimolecular Fluorescent Complementation

pSAT4-DEST-N (1–174) EYFP-C1 and pSAT5-DEST-C (175-END) EYFP-C1 vectors were used for BiFC [55]. *FLZ1* CDS and deletion fragments and PFA-DSP3 were cloned in pCR8/GW/TOPO vector and transferred to pSAT4-DEST-N (1–174) EYFP-C1 and pSAT5-DEST-C (175-END) EYFP-C1 vectors respectively using Gateway cloning technology (Invitrogen, CA). The primers used for cloning are shown in Table S3. BiFC was done in onion epidermal cells using PDS-1000 Helios Gene Gun (Biorad) [56]. Interaction was checked in TCS SP2 (AOBS) laser confocal scanning microscope (Leica Microsystems) 24 hours after bombardment.

### DAPI Staining

Onion peels were subjected to DAPI staining before visualization in confocal scanning microscope. Onion peels were washed with PBS, pH 7.5 and stained with 15 µg/mL DAPI solution for 30 minutes in dark. Peels were again washed with PBS, pH 7.5 and visualized under confocal scanning microscope.

### Subcellular Localization Study

Subcellular localization studies were done in onion epidermal cells. FLZ1 and PFA-DSP3 were cloned in pEG104 vector [57]. The constructs were bombarded in to onion peel using PDS-1000 Helios Gene Gun (Biorad) [56]. The results were analyzed 24 hours after bombardment under TCS SP2 (AOBS) laser confocal scanning microscope (Leica Microsystems).

## Supporting Information

Figure S1 **Relationship between the members of TRASH clan (CL0175).**
(PPT)

Figure S2 **Phylogenetic tree of** ***A. thaliana*** **FLZ domain containing proteins.**
(PPT)

Figure S3 **Expression profile of 3 selected FLZ domain containing genes of** ***Arabidopsis*** **.**
(PPT)

Figure S4 **Sample result of threading of FLZ domain using Phyre.**
(PPT)

Table S1 **List of FLZ domain containing proteins in sequenced plant genomes.**
(XLSX)

Table S2 **List of FLZ1 interacting proteins obtained in yeast two hybrid assay.**
(DOCX)

Table S3 **Primers used in this study.**
(DOC)

## References

[1] The *Arabidopsis* Genome Initiative (2000) Analysis of the genome sequence of the flowering plant *Arabidopsis thaliana* . Nature 408: 796–815. 11130711 10.1038/35048692

[2] Lamesch P, Berardini TZ, Li D, Swarbreck D, Wilks C, et al (2012) The *Arabidopsis* Information Resource (TAIR): improved gene annotation and new tools. Nucleic Acids Res 40: D1202–D1210. 22140109 10.1093/nar/gkr1090 PMC3245047

[3] Finn RD, Tate J, Mistry J, Coggill PC, Sammut SJ, et al (2008) The Pfam protein families database. Nucleic Acids Res 36: D281–D288. 18039703 10.1093/nar/gkm960 PMC2238907

[4] Prakash A, Yogeeshwari S, Sircar S, Agrawal S (2011) Protein domain of unknown function 3233 is a translocation domain of autotransporter secretory mechanism in gamma proteobacteria. PLoS One 6: e25570. 22073138 10.1371/journal.pone.0025570 PMC3206015

[5] Dlakić M (2006) DUF283 domain of Dicer proteins has a double-stranded RNA-binding fold. Bioinformatics 22: 2711–2714. 16954143 10.1093/bioinformatics/btl468

[6] Qin H, Chen F, Huan X, Machida S, Song J, et al (2010) Structure of the *Arabidopsis thaliana* DCL4 DUF283 domain reveals a noncanonical double-stranded RNA-binding fold for protein-protein interaction. RNA 1: 474–481. 20106953 10.1261/rna.1965310 PMC2822912

[7] Jaroszewski L, Li Z, Krishna SS, Bakolitsa C, Wooley J, Deacon AM, et al (2009) Exploration of uncharted regions of the protein universe PLoS Biol. 7: e1000205. 10.1371/journal.pbio.1000205 PMC2744874 19787035

[8] He Y, Tang W, Swain JD, Green AL, Jack TP, et al (2001) Networking senescence-regulating pathways by using *Arabidopsis* enhancer trap lines. Plant Physiol 126: 707–716. 11402199 10.1104/pp.126.2.707 PMC111161

[9] He Y, Gan S (2004) A novel zinc-finger protein with a proline-rich domain mediates ABA-regulated seed dormancy in *Arabidopsis* . Plant Mol Biol 54: 1–9. 15159630 10.1023/B:PLAN.0000028730.10834.e3

[10] *Arabidopsis* Interactome Mapping Consortium (2011) Evidence for network evolution in an *Arabidopsis* interactome map. Science 333: 601–607. 21798944 10.1126/science.1203877 PMC3170756

[11] Reiter A, Sohal J, Kulkarni S, Chase A, Macdonald DH, et al (1998) Consistent fusion of ZNF198 to the fibroblast growth factor receptor-1 in the t(8;13)(p11;q12) myeloproliferative syndrome. Blood 92: 1735–1742. 9716603

[12] Morey L, Helin K (2010) Polycomb group protein-mediated repression of transcription. Trends Biochem Sci 35: 323–332. 20346678 10.1016/j.tibs.2010.02.009

[13] Beuchle D, Struhl G, Müller J (2001) Polycomb group proteins and heritable silencing of Drosophila Hox genes. Development 128: 993–1004. 11222153 10.1242/dev.128.6.993

[14] Köhler C, Villar CB (2008) Programming of gene expression by Polycomb group proteins. Trends Cell Biol 18: 236–243. 18375123 10.1016/j.tcb.2008.02.005

[15] Zhang H, Christoforou A, Aravind L, Emmons SW, van den Heuvel S, et al (2004) The *C. elegans* polycomb gene SOP-2 encodes an RNA binding protein. Mol Cell 14: 841–847. 15200961 10.1016/j.molcel.2004.06.001

[16] Wang R, Ilangovan U, Leal BZ, Robinson AK, Amann BT, et al (2011) Identification of nucleic acid binding residues in the FCS domain of the polycomb group protein polyhomeotic. Biochemistry 50: 4998–5007. 21351738 10.1021/bi101487s PMC3938326

[17] Lechtenberg BC, Allen MD, Rutherford TJ, Freund SM, Bycroft M (2009) Solution structure of the FCS zinc finger domain of the human polycomb group protein L(3)mbt-like 2. Protein Sci 18: 657–661. 19241375 10.1002/pro.51 PMC2760371

[18] Grimm C, Matos R, Ly-Hartig N, Steuerwald U, Lindner D, et al (2009) Molecular recognition of histone lysine methylation by the Polycomb group repressor dSfmbt. EMBO J 28: 1965–1977. 19494831 10.1038/emboj.2009.147 PMC2693881

[19] Hunter S, Jones P, Mitchell A, Apweiler R, Attwood TK, et al (2012) InterPro in 2011: new developments in the family and domain prediction database. Nucleic Acids Res 40: D306–12. 22096229 10.1093/nar/gkr948 PMC3245097

[20] Goodstein DM, Shu S, Howson R, Neupane R, Hayes RD, et al (2012) Phytozome: a comparative platform for green plant genomics. Nucleic Acids Res 40: D1178–D1186. 22110026 10.1093/nar/gkr944 PMC3245001

[21] Proost S, Van Bel M, Sterck L, Billiau K, Van Parys T, et al (2009) PLAZA: a comparative genomics resource to study gene and genome evolution in plants. Plant Cell 21: 3718–3731. 20040540 10.1105/tpc.109.071506 PMC2814516

[22] Sayers EW, Barrett T, Benson DA, Bryant SH, Canese K, et al (2009) Database resources of the National Center for Biotechnology Information. Nucleic Acids Res 37: D5–D15. 18940862 10.1093/nar/gkn741 PMC2686545

[23] The Tomato Genome Consortium (2012) The tomato genome sequence provides insights into fleshy fruit evolution. Nature 485: 635–641. 22660326 10.1038/nature11119 PMC3378239

[24] Nystedt B, Street NR, Wetterbom A, et al (2013) The Norway spruce genome sequence and conifer genome evolution. Nature 497: 579–584. 23698360 10.1038/nature12211

[25] Ettema TJ, Huynen MA, de Vos WM, van der Oost J (2003) TRASH: a novel metal-binding domain predicted to be involved in heavy-metal sensing, trafficking and resistance. Trends Biochem Sci 28: 170–173. 12713899 10.1016/S0968-0004(03)00037-9

[26] Pérez-Pérez JM, Esteve-Bruna D, González-Bayón R, et al (2013) Functional redundancy and divergence within the *Arabidopsis* RETICULATA-RELATED gene family. Plant Physiol 162: 589–603. 23596191 10.1104/pp.113.217323 PMC3668055

[27] Overvoorde PJ, Okushima Y, Alonso JM, et al (2005) Functional genomic analysis of the AUXIN/INDOLE-3-ACETIC ACID gene family members in *Arabidopsis thaliana* . Plant Cell 17: 3282–3300. 16284307 10.1105/tpc.105.036723 PMC1315369

[28] Krishna SS, Majumdar I, Grishin NV (2003) Structural classification of zinc fingers: survey and summary. Nucleic Acids Res 31: 532–550. 12527760 10.1093/nar/gkg161 PMC140525

[29] Pace CN, Scholtz JM (1998) A helix propensity scale based on experimental studies of peptides and proteins. Biophys J 75: 422–427. 9649402 10.1016/s0006-3495(98)77529-0 PMC1299714

[30] Farzadfard F, Gharaei N, Pezeshk H, Marashi SA (2008) Beta-sheet capping: signals that initiate and terminate beta-sheet formation. J Struct Biol 161: 101–110. 18006332 10.1016/j.jsb.2007.09.024

[31] Zdobnov EM, Apweiler R (2001) InterProScan-an integration platform for the signature-recognition methods in InterPro. Bioinformatics 17: 847–848. 11590104 10.1093/bioinformatics/17.9.847

[32] Jones DT, Taylor WR, Thornton JM (1992) A new approach to protein fold recognition. Nature 358: 86–89. 1614539 10.1038/358086a0

[33] Miller RT, Jones DT, Thornton JM (1996) Protein fold recognition by sequence threading: tools and assessment techniques. FASEB J 10: 171–178. 8566539 10.1096/fasebj.10.1.8566539

[34] Kadrmas JL, Beckerle MC (2004) The LIM domain: from the cytoskeleton to the nucleus. Nat Rev Mol Cell Biol 5: 920–931. 15520811 10.1038/nrm1499

[35] Wu R, Terry AV, Singh PB, Gilbert DM (2005) Differential subnuclear localization and replication timing of histone H3 lysine 9 methylation states. Mol Biol Cell 16: 2872–2881. 15788566 10.1091/mbc.E04-11-0997 PMC1142431

[36] van Koningsbruggen S, Dirks RW, Mommaas AM, Onderwater JJ, Deidda G, et al (2004) FRG1P is localised in the nucleolus, Cajal bodies, and speckles. J Med Genet 41: e46. 15060122 10.1136/jmg2003.012781 PMC1735742

[37] Onodera Y, Haag JR, Ream T, Costa Nunes P, Pontes O, et al (2005) Plant nuclear RNA polymerase IV mediates siRNA and DNA methylation-dependent heterochromatin formation. Cell 120: 613–622. 15766525 10.1016/j.cell.2005.02.007

[38] Jiao Y, Wickett NJ, Ayyampalayam S, Chanderbali AS, Landherr L, et al (2011) Ancestral polyploidy in seed plants and angiosperms. Nature 473: 97–100. 21478875 10.1038/nature09916

[39] Cannon SB, Mitra A, Baumgarten A, Young ND, May G (2004) The roles of segmental and tandem gene duplication in the evolution of large gene families in *Arabidopsis thaliana* . BMC Plant Biol 4: 10. 15171794 10.1186/1471-2229-4-10 PMC446195

[40] Datta S, Hettiarachchi C, Johansson H, Holm M (2007) SALT TOLERANCE HOMOLOG2, a B-box protein in *Arabidopsis* that activates transcription and positively regulates light-mediated development. Plant Cell 19: 3242–3255. 17965270 10.1105/tpc.107.054791 PMC2174709

[41] Crocco CD, Holm M, Yanovsky MJ, Botto JF (2010) AtBBX21 and COP1 genetically interact in the regulation of shade avoidance. Plant J 64: 551–562. 21070414 10.1111/j.1365-313X.2010.04360.x

[42] Coggill P, Eberhardt RY, Finn RD, Chang Y, Jaroszewski L, et al (2013) Two Pfam protein families characterized by a crystal structure of protein lpg2210 from *Legionella pneumophila* . BMC Bioinformatics 3 14: 265. 10.1186/1471-2105-14-265 PMC3848476 24004689

[43] Takatsuji H, Matsumoto T (1996) Target-sequence recognition by separate-type Cys2/His2 zinc finger proteins in plants. J Biol Chem 271: 23368–23373. 8798540 10.1074/jbc.271.38.23368

[44] Kubo Ki, Sakamoto A, Kobayashi A, Rybka Z, Kanno Y, et al (1998) Cys2/His2 zinc-finger protein family of petunia: evolution and general mechanism of target-sequence recognition. Nucleic Acids Res 26: 608–615. 9421523 10.1093/nar/26.2.608 PMC147284

[45] Goldstein RA (2008) The structure of protein evolution and the evolution of protein structure. Curr Opin Struct Biol 18: 170–177. 18328690 10.1016/j.sbi.2008.01.006

[46] Larkin MA, Blackshields G, Brown NP, Chenna R, McGettigan PA, et al (2007) Clustal W and Clustal X version 2.0. Bioinformatics 23: 2947–2948. 17846036 10.1093/bioinformatics/btm404

[47] Biegert A, Mayer C, Remmert M, Söding J, Lupas AN (2006) The MPI bioinformatics toolkit for protein sequence analysis. Nucleic Acids Res 34: W335–W339. 16845021 10.1093/nar/gkl217 PMC1538786

[48] Brown NP, Leroy C, Sander C (1998) MView: a web-compatible database search or multiple alignment viewer. Bioinformatics 14: 380–381. 9632837 10.1093/bioinformatics/14.4.380

[49] Schuster-Böckler B, Bateman A (2005) Visualizing profile-profile alignment: pairwise HMM logos. Bioinformatics 21: 2912–2913. 15827079 10.1093/bioinformatics/bti434

[50] Kelley LA, Sternberg MJ (2009) Protein structure prediction on the Web: a case study using the Phyre server. Nat Protoc 4: 363–371. 19247286 10.1038/nprot.2009.2

[51] Crooks GE, Hon G, Chandonia JM, Brenner SE (2004) WebLogo: a sequence logo generator. Genome Res 14: 1188–1190. 15173120 10.1101/gr.849004 PMC419797

[52] Sigrist CJA, de Castro E, Cerutti L, Cuche BA, Hulo N, et al (2009) New and continuing developments at PROSITE. Nucleic Acids Res 41: D344–377. 10.1093/nar/gks1067 PMC3531220 23161676

[53] Tamura K, Peterson D, Peterson N, Stecher G, Nei M, et al (2011) MEGA5: Molecular Evolutionary Genetics Analysis using Maximum Likelihood, Evolutionary Distance, and Maximum Parsimony Methods. Mol Biol Evol 28: 2731–2739. 21546353 10.1093/molbev/msr121 PMC3203626

[54] Winter D, Vinegar B, Nahal H, Ammar R, Wilson GV, et al (2007) An “Electronic Fluorescent Pictograph” browser for exploring and analyzing large-scale biological data sets. PLoS One 2: e718. 17684564 10.1371/journal.pone.0000718 PMC1934936

[55] Tzfira T, Tian GW, Lacroix B, Vyas S, Li J, et al (2005) pSAT vectors: a modular series of plasmids for fluorescent protein tagging and expression of multiple genes in plants. Plant Mol Biol 57: 503–516. 15821977 10.1007/s11103-005-0340-5

[56] Citovsky V, Lee LY, Vyas S, Glick E, Chen MH, et al (2006) Subcellular localization of interacting proteins by bimolecular fluorescence complementation in planta. J Mol Biol 362: 1120–1131. 16949607 10.1016/j.jmb.2006.08.017

[57] Earley KW, Haag JR, Pontes O, Opper K, Juehne T, et al (2006) Gateway-compatible vectors for plant functional genomics and proteomics. Plant J 45: 616–629. 16441352 10.1111/j.1365-313X.2005.02617.x

