# Supplementary material for: DUF581 Is Plant Specific FCS-Like Zinc Finger Involved in Protein-Protein Interaction
Source: PLoS One. 2014 Jun 5;9(6):e99074. doi: 10.1371/journal.pone.0099074 (PMC4047054; doi:10.1371/journal.pone.0099074)
Supplement: Figure S1 — Relationship between the members of TRASH clan (CL0175). (PPT) [file pone.0099074.s001.ppt]

## Slide 1
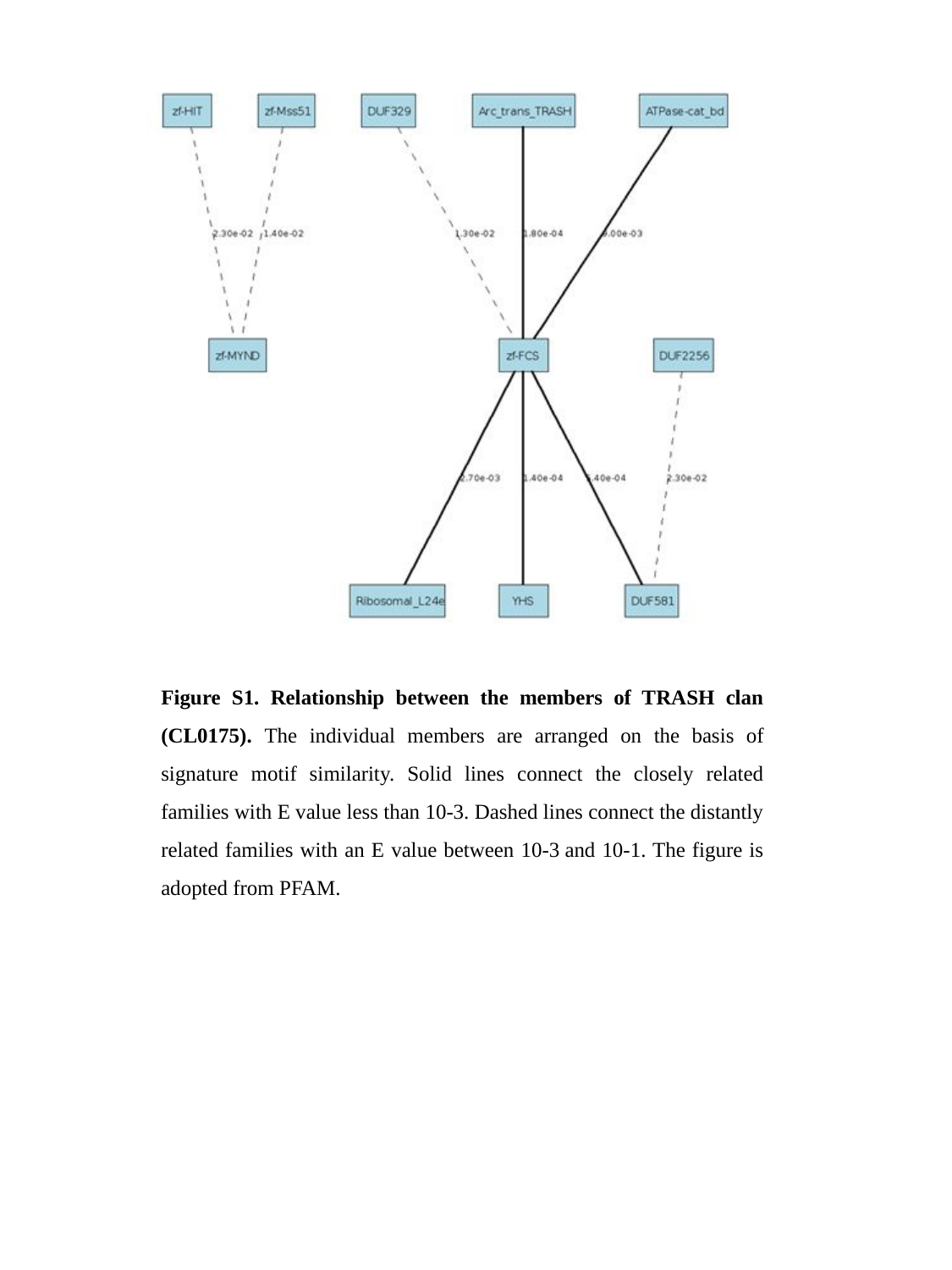

Figure S1. Relationship between the members of TRASH clan (CL0175). The individual members are arranged on the basis of signature motif similarity. Solid lines connect the closely related families with E value less than 10-3. Dashed lines connect the distantly related families with an E value between 10-3 and 10-1. The figure is adopted from PFAM.
