# Supplementary material for: DUF581 Is Plant Specific FCS-Like Zinc Finger Involved in Protein-Protein Interaction
Source: PLoS One. 2014 Jun 5;9(6):e99074. doi: 10.1371/journal.pone.0099074 (PMC4047054; doi:10.1371/journal.pone.0099074)
Supplement: Figure S2 — Phylogenetic tree of A. thaliana FLZ domain containing proteins. (PPT) [file pone.0099074.s002.ppt]

## Slide 1
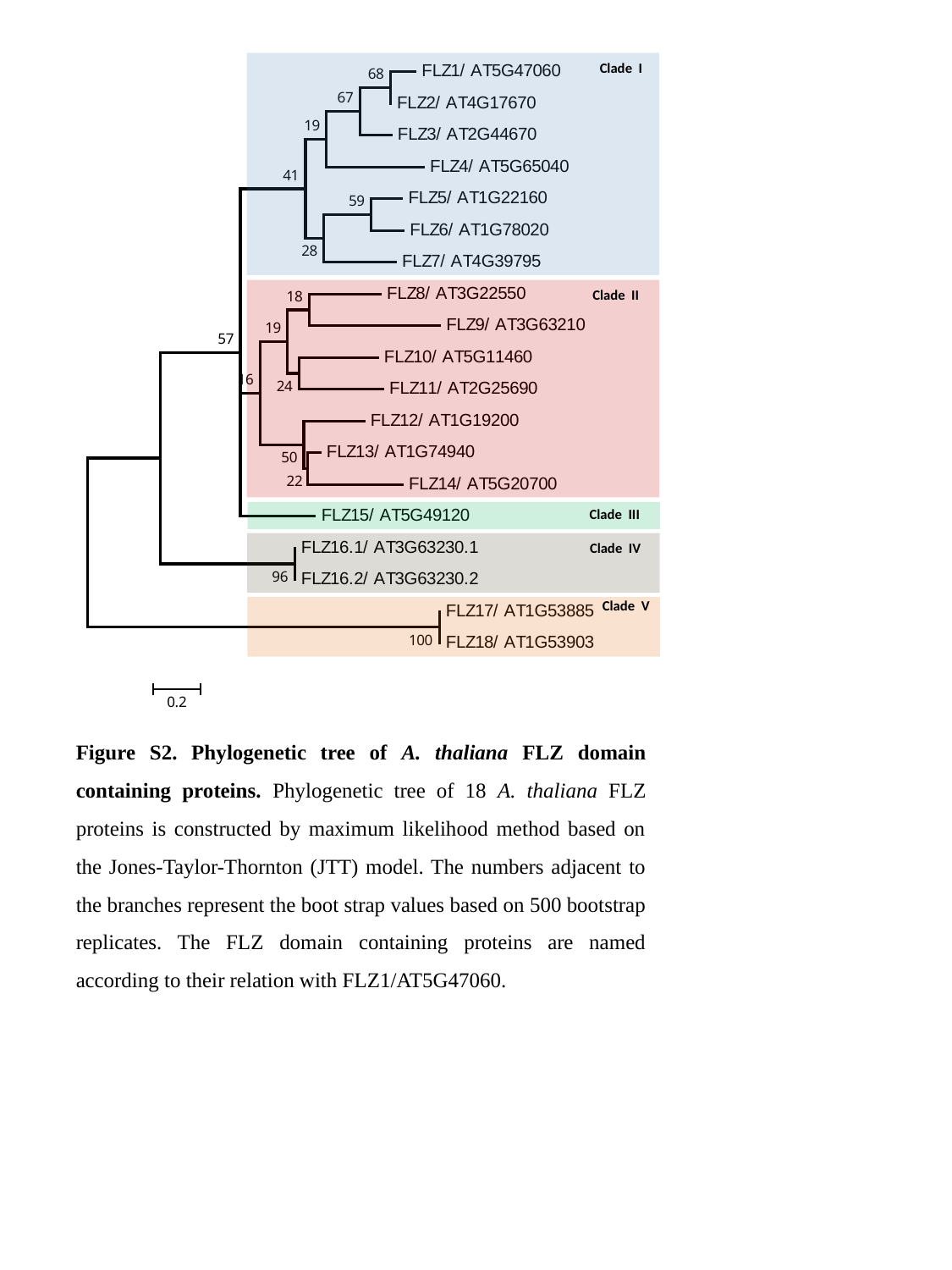

Clade I
Clade II
Clade III
Clade IV
Clade V
Figure S2. Phylogenetic tree of A. thaliana FLZ domain containing proteins. Phylogenetic tree of 18 A. thaliana FLZ proteins is constructed by maximum likelihood method based on the Jones-Taylor-Thornton (JTT) model. The numbers adjacent to the branches represent the boot strap values based on 500 bootstrap replicates. The FLZ domain containing proteins are named according to their relation with FLZ1/AT5G47060.
