# Supplementary material for: DUF581 Is Plant Specific FCS-Like Zinc Finger Involved in Protein-Protein Interaction
Source: PLoS One. 2014 Jun 5;9(6):e99074. doi: 10.1371/journal.pone.0099074 (PMC4047054; doi:10.1371/journal.pone.0099074)
Supplement: Figure S3 — Expression profile of 3 selected FLZ domain containing genes of Arabidopsis . (PPT) [file pone.0099074.s003.ppt]

## Slide 1
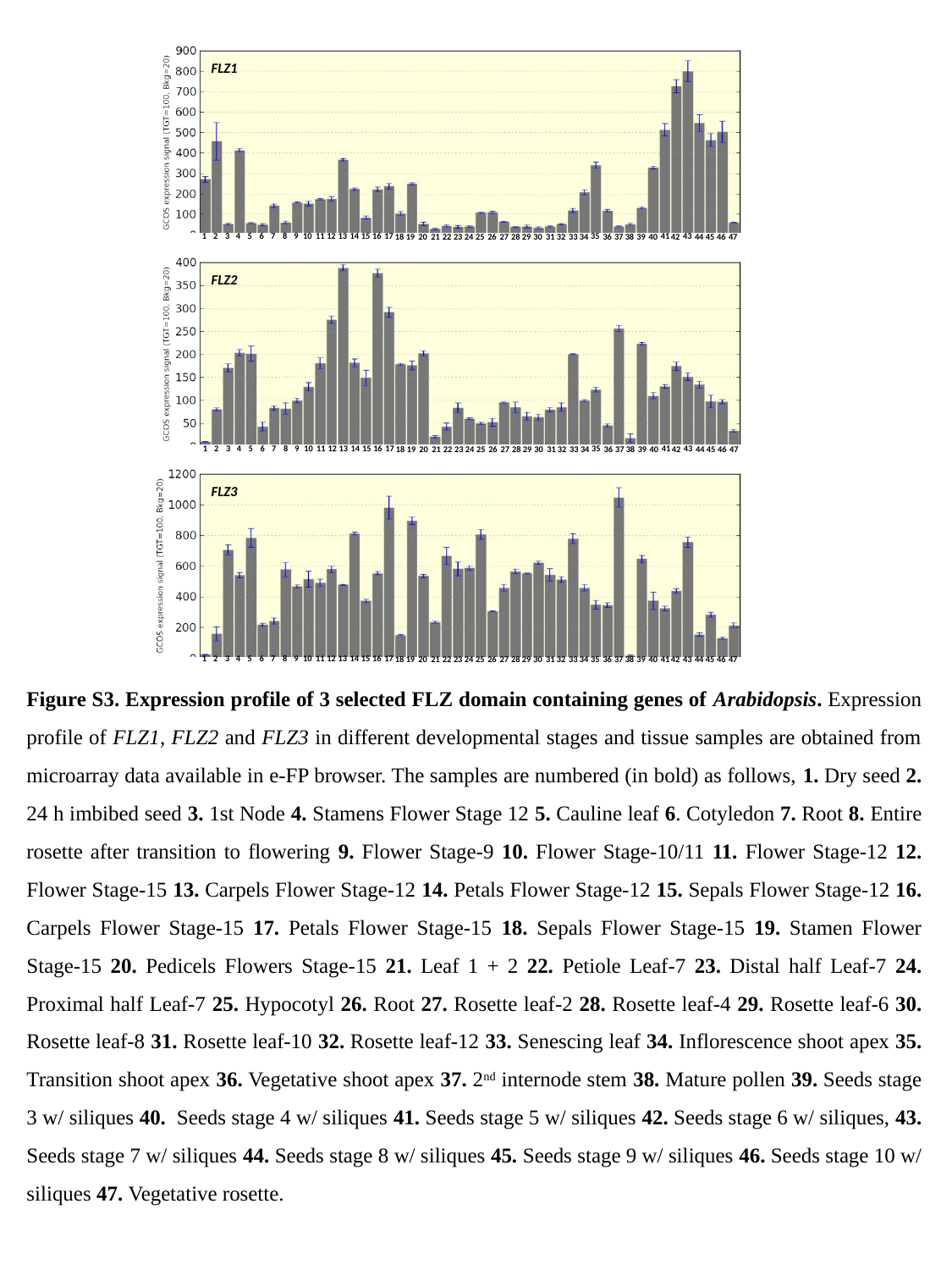

FLZ1
FLZ2
FLZ3
1
2
3
4
5
6
7
8
9
10
11
12
13
14
15
16
17
35
41
43
18
19
21
22
23
24
33
34
36
37
38
40
42
44
45
46
47
20
25
26
27
28
29
30
31
32
39
1
2
3
4
5
6
7
8
9
10
11
12
13
14
15
16
17
35
41
43
18
19
21
22
23
24
33
34
36
37
38
40
42
44
45
46
47
20
25
26
27
28
29
30
31
32
39
1
2
3
4
5
6
7
8
9
10
11
12
13
14
15
16
17
35
41
43
18
19
21
22
23
24
33
34
36
37
38
40
42
44
45
46
47
20
25
26
27
28
29
30
31
32
39
Figure S3. Expression profile of 3 selected FLZ domain containing genes of Arabidopsis. Expression profile of FLZ1, FLZ2 and FLZ3 in different developmental stages and tissue samples are obtained from microarray data available in e-FP browser. The samples are numbered (in bold) as follows, 1. Dry seed 2. 24 h imbibed seed 3. 1st Node 4. Stamens Flower Stage 12 5. Cauline leaf 6. Cotyledon 7. Root 8. Entire rosette after transition to flowering 9. Flower Stage-9 10. Flower Stage-10/11 11. Flower Stage-12 12. Flower Stage-15 13. Carpels Flower Stage-12 14. Petals Flower Stage-12 15. Sepals Flower Stage-12 16. Carpels Flower Stage-15 17. Petals Flower Stage-15 18. Sepals Flower Stage-15 19. Stamen Flower Stage-15 20. Pedicels Flowers Stage-15 21. Leaf 1 + 2 22. Petiole Leaf-7 23. Distal half Leaf-7 24. Proximal half Leaf-7 25. Hypocotyl 26. Root 27. Rosette leaf-2 28. Rosette leaf-4 29. Rosette leaf-6 30. Rosette leaf-8 31. Rosette leaf-10 32. Rosette leaf-12 33. Senescing leaf 34. Inflorescence shoot apex 35. Transition shoot apex 36. Vegetative shoot apex 37. 2nd internode stem 38. Mature pollen 39. Seeds stage 3 w/ siliques 40. Seeds stage 4 w/ siliques 41. Seeds stage 5 w/ siliques 42. Seeds stage 6 w/ siliques, 43. Seeds stage 7 w/ siliques 44. Seeds stage 8 w/ siliques 45. Seeds stage 9 w/ siliques 46. Seeds stage 10 w/ siliques 47. Vegetative rosette.
