# Supplementary material for: DUF581 Is Plant Specific FCS-Like Zinc Finger Involved in Protein-Protein Interaction
Source: PLoS One. 2014 Jun 5;9(6):e99074. doi: 10.1371/journal.pone.0099074 (PMC4047054; doi:10.1371/journal.pone.0099074)
Supplement: Figure S4 — Sample result of threading of FLZ domain using Phyre. (PPT) [file pone.0099074.s004.ppt]

## Slide 1
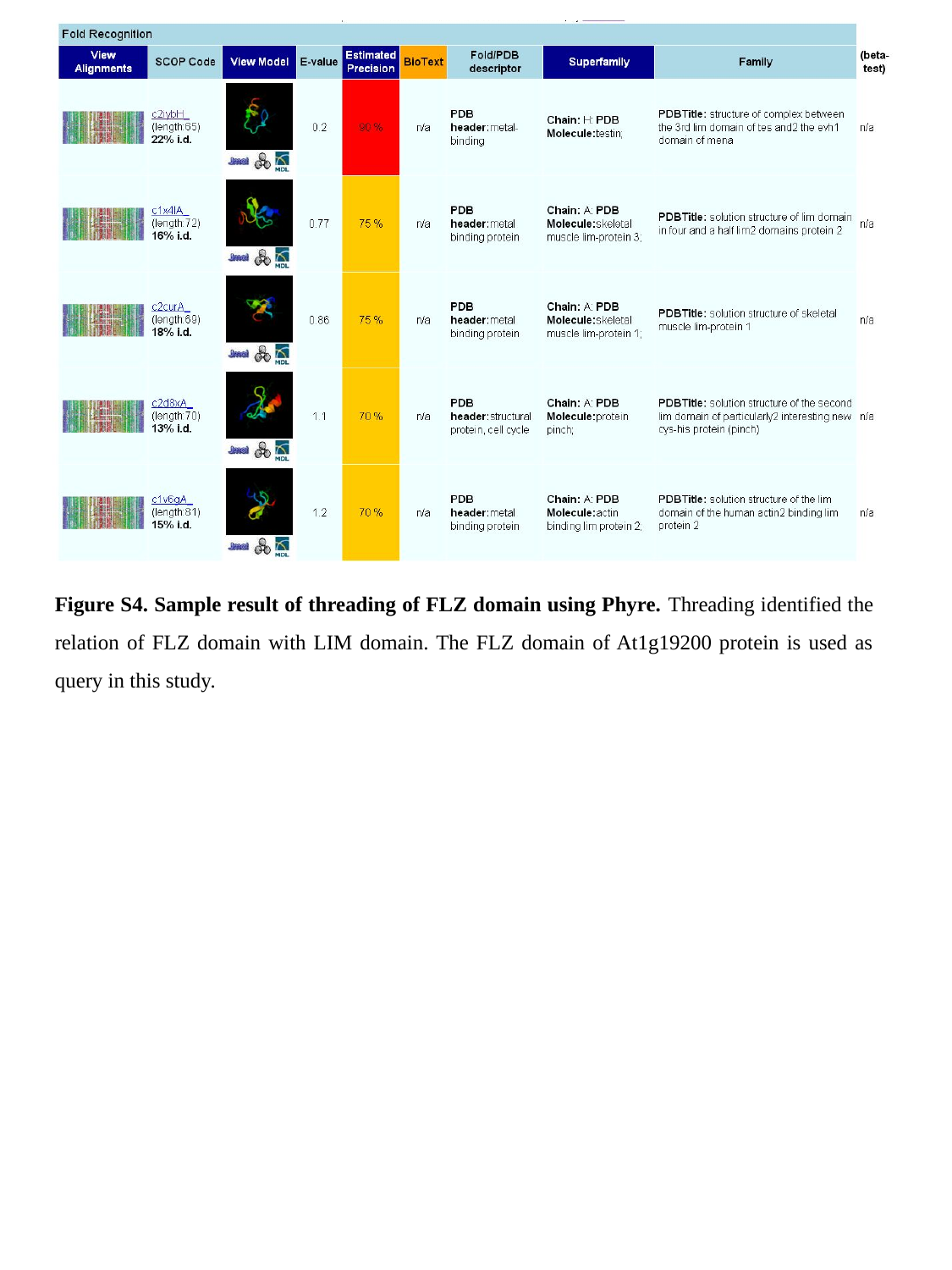

Figure S4. Sample result of threading of FLZ domain using Phyre. Threading identified the relation of FLZ domain with LIM domain. The FLZ domain of At1g19200 protein is used as query in this study.
