# Supplementary material for: DUF581 Is Plant Specific FCS-Like Zinc Finger Involved in Protein-Protein Interaction
Source: PLoS One. 2014 Jun 5;9(6):e99074. doi: 10.1371/journal.pone.0099074 (PMC4047054; doi:10.1371/journal.pone.0099074)
Supplement: Table S2 — List of FLZ1 interacting proteins obtained in yeast two hybrid assay. (DOCX) [file pone.0099074.s006.docx]

| Table S2. List of FLZ1 interacting proteins obtained in yeast two hybrid assay | |
| --- | --- |
| Name | AGI code |
| PLANT AND FUNGI ATYPICAL DUAL-SPECIFICITY PHOSPHATASE 3 | AT3G02800 |
| SALT TOLERANCE HOMOLOG 2 | AT1G75540 |
| RUBREDOXIN-LIKE SUPERFAMILY PROTEIN | AT3G15640 |
| CONSTANS-LIKE 1 | AT5G15850 |
