# Supplementary material for: DUF581 Is Plant Specific FCS-Like Zinc Finger Involved in Protein-Protein Interaction
Source: PLoS One. 2014 Jun 5;9(6):e99074. doi: 10.1371/journal.pone.0099074 (PMC4047054; doi:10.1371/journal.pone.0099074)
Supplement: Table S3 — Primers used in this study. (DOC) [file pone.0099074.s007.doc]

| **Table S3. Primers used in this study** | |
| --- | --- |
| **Name** | Sequence (5’-3’) |
| ***FLZ1* F** | CATATGATGGAGCTTTCATCGAG |
| ***FLZ1* R** | CCCGGGTTAAGCAGCAGCAAC |
| ***FLZ1* N-terminal R** | CCCGGGTTAGAAATCATGAAATC |
| ***FLZ1* domain F** | CATATGAGATTCGATATTCAACAACCTC |
| ***FLZ1* domain R** | CCCGGGTTACTCTTTTGCTTCATC |
| ***FLZ1* C-terminal F** | CATATGAAGAAACAGAATCTGTCTC |
| **TOPO-*FLZ1* F** | ATGGAGCTTTCATCGAG |
| **TOPO-*FLZ1* R** | TTAAGCAGCAGCAACAG |
| ***PFA-DSP3* F** | CCATGGATGTGTTTGATTATGGAAACG |
| ***PFA-DSP3* R** | CTGCAGTTAAACTCTAGCAGCCTGC |
| **TOPO- *PFA-DSP3* F** | CACCATGTGTTTGATTATGGAAAC |
| **TOPO- *PFA-DSP3* R** | TTAAACTCTAGCAGCCTGCG |
| ***STH2* F** | CCATGGATGAAGATCAGGTGC |
| ***STH2* R** | CTGCAGTTACCAGAAAGATCTAAAC |
| **TOPO-*AT5G49120* F** | ATGGTGGGACTAAGTATTGTTTTG |
| **TOPO-*AT5G49120* R** | GTAAGCGAAACCGCCTGC |
